# The Role of Mangroves in the Retention of Heavy Metal (Chromium): A Simulation Study in the Thi Vai River Catchment, Vietnam

**DOI:** 10.3390/ijerph17165823

**Published:** 2020-08-12

**Authors:** Anh Nguyen, Bao V.Q Le, Otto Richter

**Affiliations:** 1 Institute for Environment and Resources, Vietnam National University Ho Chi Minh City, 142 To Hien Thanh, District 10, Ho Chi Minh City 72506, Vietnam; levuquocbao.env@gmail.com; 2 Institute of Geoecology, Technical University of Braunschweig, 38106 Braunschweig, Germany; o.richter@tu-bs.de

**Keywords:** mangrove trees, heavy metal pollution, mathematical model, phytoremediation, *Rhizophora apiculata*

## Abstract

In this study, chromium (Cr) retention by the mangroves in the Thi Vai catchment located in the south of Vietnam was simulated using a coupled model of the hydrodynamic model Delft3D with Cr transport and a model for the uptake of Cr by mangroves. This coupled model was calibrated and validated using data from four hydrodynamic stations and data from phytoremediation studies. To analyze the effect of mangroves on reducing Cr pollution, three scenarios were run by the model. Scenario 1 (SC1) is based on the actual situation concerning discharges and the distribution of mangroves. Scenario 2 (SC2) simulates the deterioration of the actual situation by deforestation on the west bank and the establishment of more industrial zones on the east bank. Scenario 3 (SC3) simulates an eco-friendly development comprising the channeling of wastewater through constructed wetlands with mangroves prior to the discharge into the river. Simulation results showed that the total Cr uptake by mangroves in SC3 was higher than in the other two scenarios. In total, 33 kg Cr in water were absorbed by the constructed wetlands in SC3 within one month. The simulation results helped in overcoming the difficulties and challenges in assessing the capacity of mangrove forests on the retention of chromium at catchment scale.

## 1. Introduction

Pollution in mangrove estuaries, especially heavy metal pollution, has been extensively addressed worldwide because of its manifest adverse effects on ecosystems and human health [1]. Many researchers in recent years have focused on heavy metals concentration and their distribution, sources and fate in the estuarine environment [2,3]. Excess of sedimentary elements concentration leading to potential risk of ecosystem health is a challenge such that many efforts try to reduce and clean up pollutants in these highly dynamic environments.

Mangrove trees have the ability of storing metals, transferring these elements from the sediment and concentrating them in their tissues [4]. They can serve as a means for the immobilization and removal of pollutants. This functionality has been studied and proven to be a good practical method to treat pollutants [5]. It is said that mangrove can serve as a natural wastewater treatment system [6,7,8], which could be considered as a phytoremediation process.

Phytoremediation is a treatment method related to the use of vegetation growing on a polluted environment to either contain, remove or render toxic environmental contaminants harmless [9]. Phytoremediation has gained more attention from researchers although it is not a new concept because of its low cost compared to physico-chemical remediation systems [10].

Studies on metal transfer and accumulation in mangrove plants have been conducted intensively worldwide. The ability of mangrove to accumulate pollutants depends on the type of pollutants, plant species and environmental conditions [11,12]. Wen-jiao et al. [13] found that *Rhizophora stylosa* in Yinglou bay (China) absorbed a high concentration of cadmium (Cd) (with a high accumulation coefficient of 1.4, compared to other metals). When analyzing the removal of heavy metal by mangrove species, Fengzhong et al. [14] found that *Avicennia marina* has high removal performance of lead (Pb, 83.8%) and Cd (74.2%), *Kandelia candel* has ability to absorb copper (Cu, 70.5%) and nickel (Ni, 50.5%). MacFarlane et al. [15] conducted experiments and reported that Cu was accumulated in root tissues of *A. marina* under a certain concentration limit (200 µg/g). Linear relationships of heavy metals up taken by mangrove plants and their concentration in sediment were found. Inhibition effects on seedling height and leaf number were observed at extreme Cu concentration (800 µg/g) by [16]. *Rhizophora apiculata* in the study of Kamaruzzaman et al. [17] in Malaysia showed the potential of metal accumulation. The concentration in the root of this species was higher than in the bark and in the leaf. The mean concentrations in leaf, bark and root, respectively, were 2.73 mg/L, 3.94 mg/L and 5.21 mg/L for Cu and 1.43 mg/L, 1.38 mg/L and 2.05 mg/L for Pb. In the work of Nazli and Hashim [18], they found the accumulation of Cu and Pb in roots and leaves of *Sonneratia caseolaris* (concentrations of Pb and Cu for leaves were 35.5μg/g and 26.8 μg/g and for roots were 92.9 μg/g and 31.2 μg/g, respectively) and came to the conclusion that the roots of this species have high capacity in taking up heavy metals and could be a potential phytoremediation species for heavy metal treatment in Malaysian mangrove ecosystems. In their report on the physiological responses of *Sonneratia apetala* to heavy metal, Zhang et al. [19] conducted an experiment on pollutant treatment by plants with artificial wastewaters at different concentrations and environmental conditions. They described that no harmful symptom was found at high metal concentration conditions (Pb: 10 mg/L; Cu: 20 mg/L; Cd: 1 mg/L) and the nutrition contents in wastewater enhanced the growth of *Sonneratia apetala* [19]. Richter et al. [20] provided the phytoremediation performance of young *Rhizophora apiculata* in treating Cr in industrial wastewater and showed the maximum Cr uptake by this species (at 500 mg/L Cr in water) was 326.72 mg/kg after 3 months and 126.9 mg/kg after 6 months. Titah and Pratikno [21] discussed that *Avicennia alba* can be considered for use in the phyto-monitoring and phytoremediation of Cr in coastal areas. Chowdhury et al. [22] concluded that the species *Sonneratia apetala* and *A. officinalis* are potential hyperaccumulators which accumulate high concentrations of Cu (25.89 mg/kg), Fe (1376.7 mg/kg) and Cr (2.85 mg/kg) in the pneumatophore. They also concluded that *Rhizophoraceae* and *R. apiculata* are potential accumulators for Hg and Pb.

However, in these laboratory and greenhouse experiments, spatial heterogeneity concerning soil properties and initial contaminant levels is kept low, which is usually not the case in the field. The uneven distribution of contaminants is a frequently encountered challenge in field studies, which results in data scatter. This is also problematic in terms of regulatory standards because remediation success is often judged on a point-by-point basis, rather than an average of data points from across the site [23].

There is a large potential in the application of mathematical models to simulate those processes, because a large amount of field data and even data from experiments are available. There exist some mathematical models for phytoremediation processes. Burken and Schnoor [24] developed a mathematical model to describe the physical, chemical and biological mechanisms of the phytoremediation of poplar trees from experimental data. Chiou et al. [25] developed a mathematical model for phytoremediation, taking into account environmental factors. Shashi [26] simulated the phytoremediation of Cd by plants using the system of solute transport equations, coupled with the one-dimensional Richard equation with a root extraction term. For modelling the phytoremediation by mangroves, Richter et al. [20] developed a phytoremediation model for the mangrove species *Rhizophora apiculata* based on experimental data. There are only few models for the retention of heavy metals by mangroves at catchment scale. The approach of Nguyen and Richter [27] showed that a coupling of hydrodynamics with an environmental fate model for chromium in the soil-plant domain is feasible; however, this model was at the theoretical stage and was not calibrated for a real situation.

Our interest in this study was to develop a mathematical model and apply this model to predict the phytoremediation of mangroves from an uneven distribution of contaminants in the field using the available scattered data. This model can help to: (i) calculate the amount of heavy metal taken up by mangrove forests at landscape level; (ii) evaluate the different alternatives of management regimes in mangrove areas; (iii) support the design of a phytoremediation system in the field. The objective of the approach presented in our paper is to close the gap between theoretical concepts and application to a real environmental problem in a catchment. This was achieved by model calibration and validation using available field and experimental data in the Thi Vai River (data obtained from the previous work of Nguyen [28], Richter et al. [20] and Costa-Boddeker et al. [29]). The concept was realized by implementing a compartment model for substance transport in the soil-plant domain into the hydrodynamic model Delft3D for riverine transport. This model was used to simulate the distribution of chromium in water, soil and mangrove trees for different scenarios of environmental management.

Chromium (Cr) concentrations were detected mainly in our study area, the Thi Vai catchment, which is located in the South of Vietnam. Thi Vai river separates the Can Gio mangrove forest in the West and the industrial area in the East. This river and its surroundings have been severely polluted because of the direct discharge of wastewater from the industrial companies. Nguyen [28] found high concentrations of heavy metals, especially chromium, accumulated in the soils and in different organs of mangrove trees along this river (the highest concentration in the surface soil was 634 mg/kg and in tree root was 90.6 mg/kg). In the aquatic environment, Cr has two main oxidation states—Cr(III) and Cr(VI). Cr(VI) is considered as a highly toxic compound due to its solubility, oxidizing potential and ability to cross cell membranes [30]. In our study, Cr oxidation forms were not specified; however, in general, both speciation forms—Cr(III) and Cr(VI)—occur in this area. Costa-Boddeker et al. [29] reported that measured Cr concentrations in Thi Vai water are much higher compared to other estuarine areas in Asia and tropical estuaries in Brazil (mean values of 0.01 mgL^-1^). It is about six-fold higher than those recorded by Kumar et al. [31] in Victoria Bay, Southeast Brazil. Cr is extensively used by the industries located in the Thi Vai catchment, such as textile manufacturing, electroplating and tanneries, among others, which dispose wastewater to the Thi Vai river. The tanning industry has been considered as one of the most polluting industries in Vietnam. About 65% of the tanneries are not in accordance with the Vietnamese Standard on industrial wastewater because most of them ignored the regulation to install a wastewater treatment system [29]. In our study area several tanneries are located along the Thi Vai river and might contribute to Cr concentrations in the water. The pollution of coastal waters with Cr(III) and Cr(VI) has been associated with tannery operations worldwide (e.g., Gardner and Ravenscroft [32], Suprapti et al. [33], Abdulla et al. [34]).

## 2. Integrated Numerical Model

### 2.1. Compartment Model for Pollutant Transport in the Water–Soil–Plant Domain

**Figure 1:**
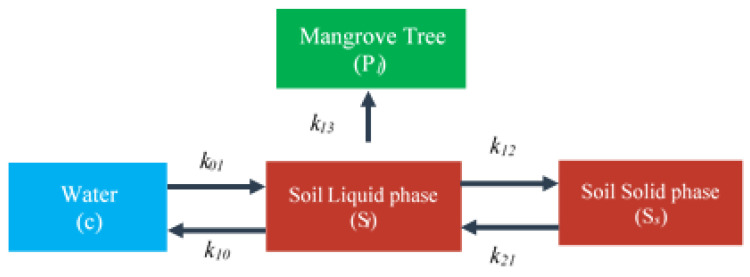
Compartment scheme for matter transport in the water–soil–plant domain; parameters *k_ij_* are kinetic constants.

A simplified version of the compartment model given by our previous work [27] was developed, comprising concentrations in water, in the liquid and solid phases of the soil and in the mangrove tree, as shown in Figure 1. The plant domain was not further differentiated into single plant organs as it was in our model before [27].

At landscape scale, it is necessary to bring together a transport model of Cr in a real river system and an environmental fate model for Cr in the soil-plant system. This involves the coupling of partial differential equations (transport) with a system of ordinary differential equations. For the water domain, we applied the two-dimensional shallow water equations coupled with the convection diffusion equation for pollutant transport and reaction using Delft3D source codes. Delft3D was developed by the Netherlands WL, Delft Hydraulics. Delft3D-Flow is one of the most widely used hydrodynamic models for river flow simulation (e.g., in Javernick et al. [35], Williams et al. [36]) and for water quality simulation (e.g., in Duy Vinh et al. [37]). Its performance compares well with other systems (e.g., with Mike 21 [38]). Furthermore, Delft3D is an open source software. The model is capable of predicting the hydraulic phenomena in the simulated river with coherent results according to the governing system of partial differential equations. The state variables of soil and plant compartments were embedded into the Delft3D code. The coupling is mediated via the concentrations in the water, *c*(*x*,*y*). The embedment of the soil-plant compartment model into the hydrodynamics model Delft3D was conducted using the programming software Matlab R2018b.

Assuming linear kinetics, the mass balance equations for Cr in three compartments are given by

(1) $$\frac{\partial S_{l}}{\partial t}=k_{01}c-\left(k_{12}+k_{13}+k_{10}\right)S_{l}+k_{21}S_{s}$$

(2) $$\frac{\partial S_{s}}{\partial t}=k_{12}S_{l}-k_{21}S_{s}$$

(3) $$\frac{\partial Pl}{\partial t}=k_{13}S_{l}$$

where: *c*—Cr in water; *S_l_*—Cr in soil liquid phase; *S_s_*—Cr in soil solid phase; *P_l_*—Cr in plant; *k_i,j_*—kinetic constants. Note that the concentration in the water, *c*, is provided by the transport Equation (10).

### 2.2. Hydrodynamics and Water Quality Models Delft3D

As described above, to deal with the complexity of the fate of pollutants in the environment’s water, soil and plant, the two modules of hydrodynamics and water quality from Delft3D were applied to simulate the transportation of matter in a water environment (water domain in the compartment model described in Figure 1).

In the hydrodynamic module Delft3D-Flow, numerical solutions of the unsteady state shallow water equations in two (depth averaged) or three dimensions are provided based on the finite element method. In this approach, the vertical momentum equation is reduced to the hydrostatic pressure relation. Vertical accelerations are assumed to be small compared to gravitational acceleration and are not taken into account. The governing equations given by Luijendijk [39] are the continuity equation:

(4) $$\frac{\partial \zeta }{dt}+\frac{\partial \left(d+\zeta \right)u}{\partial x}+\frac{\partial \left(d+\zeta \right)v}{\partial y}=0$$

and the momentum equation:

(5) $$\frac{\partial u}{\partial t}+u\frac{\partial u}{\partial x}+v\frac{\partial u}{\partial y}+g\frac{\partial \zeta }{\partial x}-fv+\frac{\tau_{bx}}{\rho_{w}\left(d+\zeta \right)}-\frac{F_{x}}{\rho_{w}\left(d+\zeta \right)}-K\left(\frac{\partial^{2}u}{\partial x^{2}}+\frac{\partial^{2}u}{\partial y^{2}}\right)=0$$

(6) $$\frac{\partial v}{\partial t}+u\frac{\partial v}{\partial x}+v\frac{\partial v}{\partial y}+g\frac{\partial \zeta }{\partial x}+fu+\frac{\tau_{by}}{\rho_{w}\left(d+\zeta \right)}-\frac{F_{y}}{\rho_{w}\left(d+\zeta \right)}-K\left(\frac{\partial^{2}v}{\partial x^{2}}+\frac{\partial^{2}v}{\partial y^{2}}\right)=0$$

where *d*—water depth (m); ζ—water level above the horizontal plane of reference (datum) (m); *f*—Coriolis parameter (1/s); *F_x_, F_y_*—x-, y- components of external forces (N/m^2^); *u,* v—fluid velocities in the x- y- directions (m/s); $\rho_{w}$—mass density of water (kg/m^3^); K—eddy viscosity (m^2^/s); g—gravity of acceleration (m/s^2^); $\tau_{bx,y}$—x- y- components of bed shear stress (N/m^2^).

The bottom roughness in the Delft3D-FLOW program can be defined in several ways. For a depth-averaged flow (2D), the shear stress on the bed in the x- and y-direction induced by a turbulent flow is given by a quadratic friction law:

(7) $$\tau_{bx}=\rho_{w}g\left(\frac{\left|U\right|u}{C^{2}}\right)$$

(8) $$\tau_{by}=\rho_{w}g\left(\frac{\left|U\right|v}{C^{2}}\right)$$

The 2D-Chézy coefficient C depends on the Manning coefficient *n*:

(9) $$C=\frac{\sqrt[{6}]{h}}{n}$$

where U—absolute magnitude of total velocity; $U={\left(u^{2}+v^{2}\right)}^{1/2}\left(m/s\right)$; h—total water depth (m).

Delft3D also came with a water quality module (D-WAQ) for modeling and predicting the transportation of pollutants in water (in this case Cr). The depth-averaged pollutant transport in this two-dimensional model is given by the advection–dispersion equation:

(10) $$\frac{\partial C}{\partial t}+\upsilon_{x}\frac{\partial C}{\partial x}-D_{x}\frac{\partial^{2}C}{\partial x^{2}}+\upsilon_{y}\frac{\partial C}{\partial y}-D_{y}\frac{\partial^{2}C}{\partial y^{2}}=F\left(x,y,t\right)-k_{01}c+k_{10}s_{l}$$

where $D_{x,y}$—dispersion coefficient in x, y direction (m^2^/s); υ—velocity (m/s); c—concentration in water (g/m^3^); F(x,y,t)—discharge source; *k_01,_ k_10_*—kinetic constants. Note that this equation is coupled with the compartment model via the last two terms.

## 3. Site Description

### 3.1. Study Area

**Figure 2:**
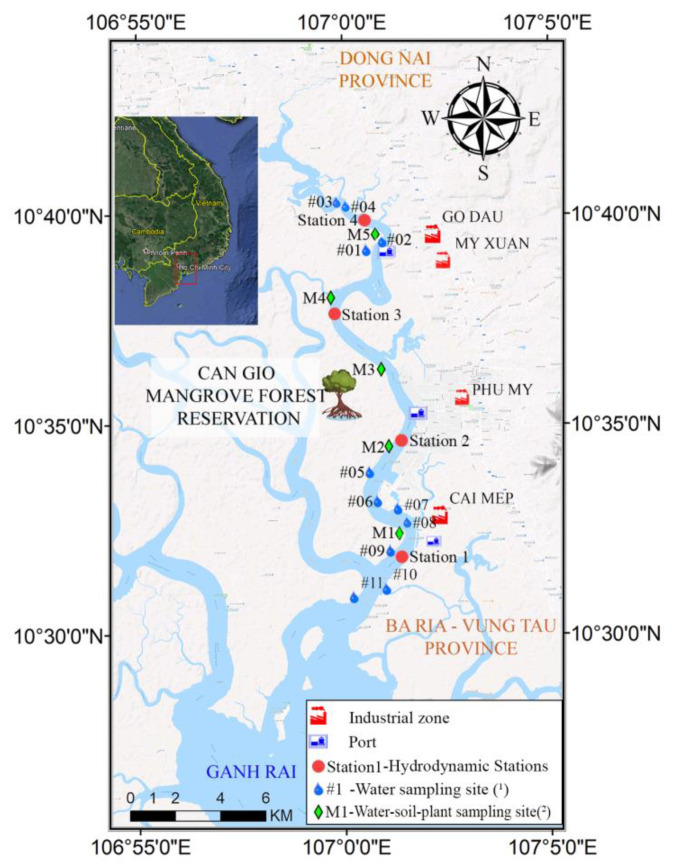
The Thi Vai catchment and the sampling sites (basemap is from Google Earth Terrain map 2018).

The Thi Vai River is located in the estuary of the Dong Nai–Sai Gon River system in the south of Vietnam, as shown in Figure 2, from 10°28′ N to 10°39′ N and 107°00′ E to 107°14′ E. The total length of the river is approximately 76 km with an average depth of 10–20 m, the maximum depth is about 60 m. The hydrological regime in the area is dominated by semi-diurnal tidal regimes with high amplitude. The Thi Vai river was chosen as our study area because this river is one of the most polluted rivers in Vietnam. The area at the East bank of this river has been highly industrialized in the past two decades—more than 14 industrial zones and international ports were established. Uncontrolled wastewater was discharged directly to the river from industrial activities. Costa-Boddeker et al. [29] found that the sediments in this river catchment have been heavily polluted by heavy metals. Chromium was found to be one of the most enriched heavy metals in this area [40]. Furthermore, the catchment is adjacent to the Can Gio Biosphere Reserve, which has been listed as a biosphere reserve by UNESCO. In addition, bathymetry and discharge data, a digital elevation model and chromium concentration data were available for this catchment.

### 3.2. Bathymetry Data

**Figure 3:**
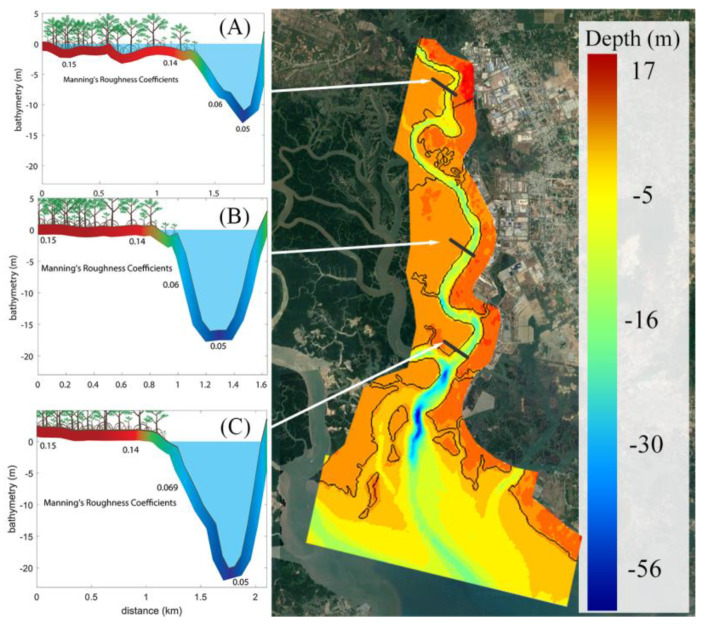
Bathymetry of the study area and distribution of Manning coefficient values at upstream (**A**), middle stream (**B**) and downstream (**C**) locations.

Bathymetry data for our calculation were obtained from different sources, including: (1) high resolution (10 × 10 m) bathymetric data of the river bed collected at the Southern Vietnam Maritime Safety Corporation; (2) 100 × 100 m resolution bathymetric data at the Ganh Rai Bay collected at Southern Vietnam Maritime Safety Corporation; (3) land elevation (50 × 50 m) of the surrounding areas collected at the Institute for Environment and Resources to produce the topography of the surrounded areas of the Thi Vai River, including flood plains and islands (areas where other bathymetry sources have no coverage). The bathymetry data from all the above sources were combined to construct a continuous bathymetry for the main channel and adjacent flood plain with a resolution ranging from 10 to 50 m, as shown in Figure 3. These bathymetry data were interpolated onto the 2D computational grid (in DELFT3D model) to construct the required model geometry.

### 3.3. Hydrometric and Tide Data

The hydrometric data (discharge and water level) were obtained from the Southern Institute of Water Resources Research (SIWRR), including hourly hydrometric data (measured for 15 days, in dry and rainy seasons) at four stations located along Thi Vai River, as shown in Figure 2. According to this data, the upstream of the Thi Vai River provided a limited freshwater flow with average discharges of 580 m^3^/s in the dry season and 647 m^3^/s in the rainy season. At open boundaries, the astronomical components of the tide data were obtained from the Global Inverse Tide model (TPXO 7.2). The Global Inverse Tide model (TPXO 7.2) was chosen since it gave the best result for our model performance. Gladkikh and Tenzer [41] compared several global ocean tide models and came to the conclusion “We have assessed the regional accuracy of the global ocean tide models TPXO7.2, GOT00.2, NAO.99b, FES2004, and EOT10a in New Zealand using the tide-gauge data from Wellington, Tauranga, Taranaki, Marsden Point, Jackson Bay, Timaru, and Port Charmers. The accuracy analysis revealed that TPXO7.2 provides the best solution for this part of the world”. The average tidal amplitude in our study area was 2.7 m. These data were used to set up the boundary conditions, calibration and validation for the DELFT3D hydrodynamic model.

### 3.4. Data of Chromium Concentrations in Water–Soil—Plant Domains

Data of chromium concentrations in water–soil–plant domain in this region were collected from two sources. Firstly, experimental data from our previous work [20,42]. Richter et al. [20] and Nguyen et al. [42] conducted experiments to examine the functioning of a pilot phytoremediation system of young mangrove species, *Rhizophora apiculata,* in dependence on pollutant loads and other environmental variables. In this experiment, young mangrove trees were planted in pots placed into aquaria, each containing 6 kg of soil. The soil was taken from inside the mangrove forest in the Thi Vai catchment. Concentrations of Cr in water, soil and plant were analyzed at the beginning of the experiment, at month three and then at month six during the experiment. For more details of this experimental design, see [20]. Secondly, our field sampling data were taken in January 2013 at the sampling sites along the river, as shown in Figure 2, and were already published [27,29]. Heavy metals in all samples were analyzed and Cr was found with high concentrations in all samples of water–soil–plant, especially at the middle to upstream sites of the river. These data were used to calibrate and validate our compartment model for substance transport in water–soil–plant media (described in Section 2).

## 4. Model Setting and Scenarios

The 2D domain for the numerical simulation extends about 25 km from upstream to downstream of the Thi Vai River, comprising the main river channel and the floodplain. The bathymetry data which are described in Section 3.2 were interpolated onto the 2D Cartesian grid of 27,901 cells with resolutions ranging from 60 m^2^ at upstream to 85 m^2^ at the Ganh Rai bay boundary. The dimension of the grid matrix was 282 × 240 cells.

Upstream open boundary conditions were taken from the hydrological data at station four, as shown in Figure 2. Downstream boundary conditions are sea open boundaries and were adapted from the Global Inverse Tide model (TPXO 7.2) of the same period. Simulation time was set up for one month in January 2013 to suit observational data.

### 4.1. Calibration and Validation for the Model

The coupled model needs to be calibrated and validated before it is applied to run for different scenarios. The process of calibration and validation for the model was conducted in two steps. The first step was the calibration and validation process for the hydrodynamic model, based on bathymetric and tide data from four stations located along the river. The second step was the parameter estimation for the compartment model for Cr transport in the water–soil–plant domain based on data from our previous phytoremediation studies.

Manning coefficients are spatially distributed and depend on the presence of soil and mangrove vegetation. We applied the Manning coefficient values from the works of [43,44]. The values of this coefficient were 0.005 for the riverbed and 0.15 for the mangrove flood plain [43,44]—see Figure 3. The Manning coefficients used in our study are frequently found in the literature. For example, McIvor et al. [45] reported the Manning values for different land use types, which correspond closely to our values.

**Figure 4:**
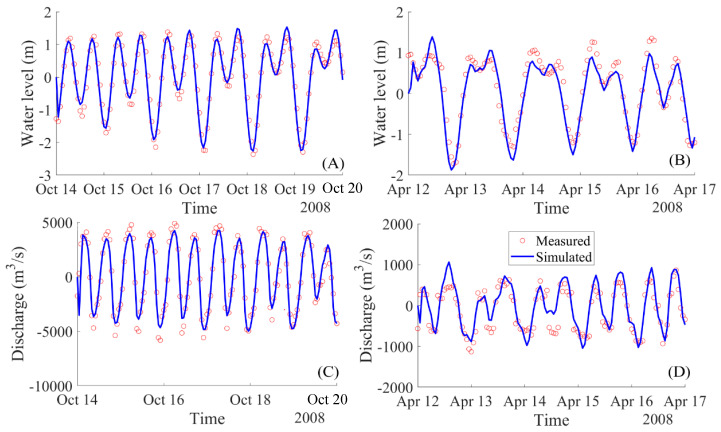
Calibration phase. Computed and measured water levels and discharges at the representative stations in rainy season (station one: (**A**,**C**)) and in dry season (station four: (**B**,**D**)).

**Figure 5:**
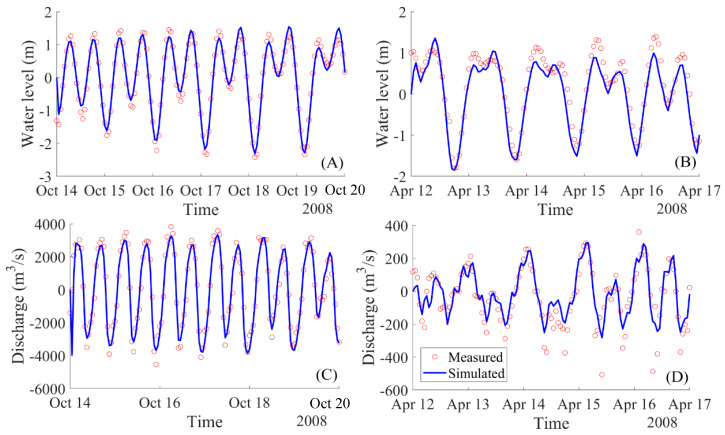
Validation phase. Computed and measured water levels and discharges at the representative stations in rainy season (station two: (**A**,**C**)) and in dry season (station three: (**B**,**D**)).

Calibration of the horizontal eddy viscosity was carried out by simulating the estuarine hydrodynamics with different coefficient values in the range [0,5] (m^2^/s), which is frequently found in the literature (e.g., Parsapour-Moghaddam et al. [46]). In our case the value of 1 m²/s gave the best fit. Roy et al. [47] also reported a value of 1 m²/s. This calibration process was conducted using the data from stations one and four for both dry and rainy seasons, as shown in Figure 4. The other two stations—two and three, as shown in Figure 5—were used for validating the model to verify the model accuracy after setting the model parameters.

**Figure 6:**
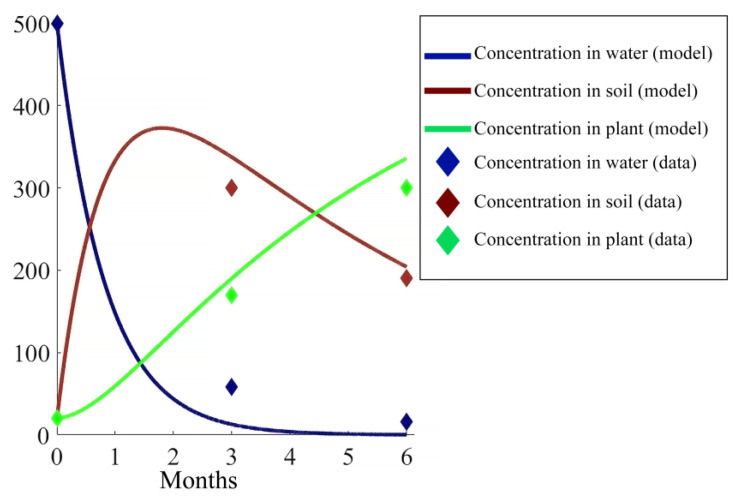
Model fit to greenhouse experimental data. Concentration in water is in mg/L and concentration in soil and plant is in mg/kg.

For the compartment model for Cr transport in the water–soil–plant domain, the starting point for the calibration of the model were the parameter values from the experiments of Richter et al. [20] and Nguyen et al. [42] conducted in a constructed wetland involving young mangrove plants. The model fit to a data set of the experiment is shown in Figure 6. These parameters values were applied in the model for the artificial wetlands in scenario three with young mangroves. However, the mangroves in the Thi Vai area comprise an even aged mature forest which was planted during the period 1990–1993 and the uptake ability of the mature tree is different from the young tree. Thus, the parameter k_13_ of the model (which describes the uptake of Cr from soil to mangroves and depends on tree age and size) had to be adapted to the actual field situation by tuning. Field data of Cr concentrations in water, soil and plant were used as initial conditions and Cr loads were taken from the discharge data of the industrial sites.

To evaluate the performance of the model, the following three efficiency criteria were used:

Nash–Sutcliffe efficiency (NSE) [48]: NSE = 1 corresponds to a perfect match of model prediction to the observed data. NSE = 0 indicates that the model predictions are as accurate as the mean of the observed data, whereas an efficiency less than zero (NSE < 0) occurs when the observed mean is a better predictor than the model. Threshold values to indicate a model of sufficient quality have been suggested between 0.5 < NSE < 0.65.

Index of agreement (d): A standardized measure of the degree of model prediction error which varies between 0 and 1. The index of agreement represents the ratio of the mean square error and the potential error. An agreement value of 1 indicates a perfect match, and 0 indicates no agreement at all [49,50].

Root mean square error (RMSE) [51]: RMSE is the square root of the average of squared errors. RMSE is always non-negative, and a value of 0 (almost never achieved in practice) would indicate a perfect fit to the data. In general, a lower RMSE is better than a higher one.

### 4.2. Scenarios Description

**Figure 7:**
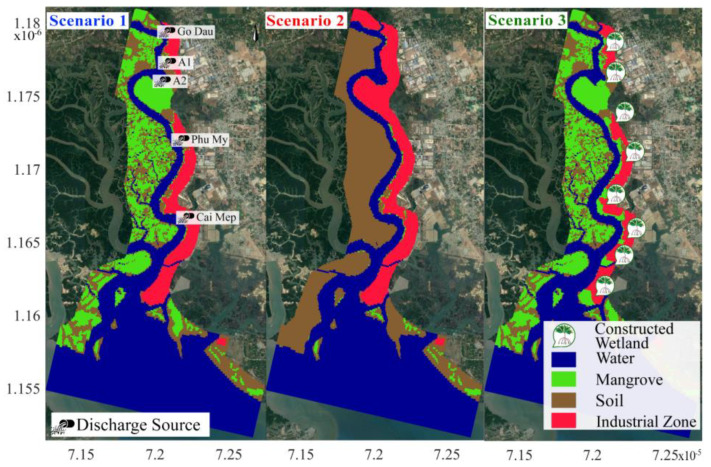
Maps of situations of three scenarios (background image is from Google Earth, 2018).

To better understand the efficiency of mangroves in treating heavy metal pollutants, we applied this calibrated and validated model to run three scenarios of different management strategies. The first scenario (SC1) is the baseline scenario which reflects the current situation of mangrove distribution and industrial activity. Scenario two (SC2) comprises the destruction of mangrove forests on the West bank of the river and the establishment of more industrial zones on the East bank. Scenario three (SC3) describes an eco-friendly development with the following features: a series of constructed mangrove wetlands is established on the East bank; industrial wastewater is channeled through these wetlands prior to the discharge into the river; mangrove forests still exist on the West banks. Figure 7 shows the three described scenarios. Wastewater discharges and locations, as shown in Table 1, from industrial companies, are set to be similar in all scenarios. The initial concentration of Cr in the Thi Vai River was set to zero at the start of every simulation to facilitate the comparison.

## 5. Results and Discussion

### 5.1. Results of Model Calibration and Validation

#### 5.1.1. Calibration and Validation for the Hydrodynamic Model Delft3D

The comparison of temporal variation between the measured and simulated water levels at stations one and four is shown in Figure 4. These stations are located at 9 km (downstream location, station one) and 22 km (upstream location, station four) from the Ganh Rai Bay, as shown in Figure 2 for station one and four. The predicted water levels and water discharges were reasonably matched with the measured data at these two monitoring stations. The tidal asymmetry observed at the two stations was reasonably reproduced by the model. Regression analysis was carried out for the measured and simulated water levels at the two stations. The model successfully reproduced the water level in the river in both amplitude and phases with NSE and the d values are larger than 0.8 and 0.9 for these two locations, respectively, and the RMSE is smaller than 0.4. These indices values are presented in Table 2.

For the validation process, the statistical analysis to verify model performance is shown in Table 2 using data from the stations two and three, as shown in Figure 5. The results showed that simulated water levels were in acceptable agreement with the measured water level for the two validated stations, two and three, and at two seasons with NSE > 0.6, d > 0.89 and RMSE <1.

For the simulation results of water discharge, there remain larger discrepancies from the results for the case in the dry season than for the case of data in the rainy season at the peak values of the flows, as shown in Figure 4D and Figure 5B,D. However, these error values are acceptable, as is shown in the model performance measures in Table 2. This problem was discussed by many authors, such as N. Moriasi et al. [52] or Krause et al. [51]. Performance can be improved for future studies by using the higher spatial resolution of the bathymetry.

#### 5.1.2. Parameters Estimation for the Compartment Model

**Figure 8:**
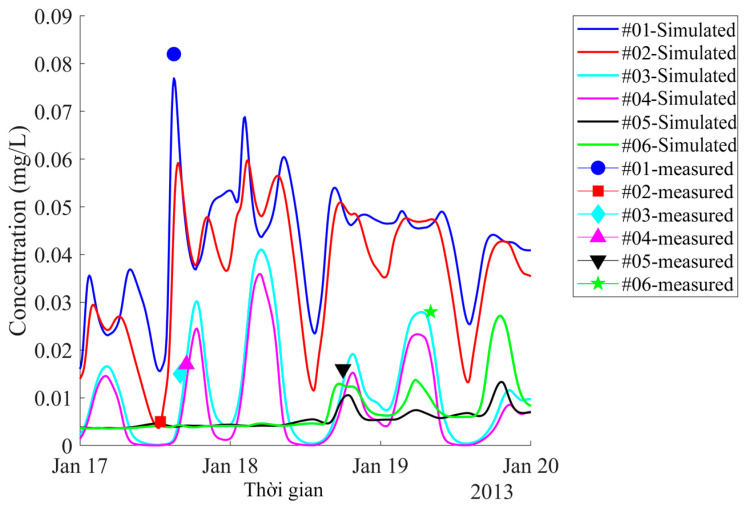
Simulated time course of concentration of chromium in water at stations 1–6 located at industrial zones and measured data. Locations of these stations are shown in Figure 2.

**Figure 9:**
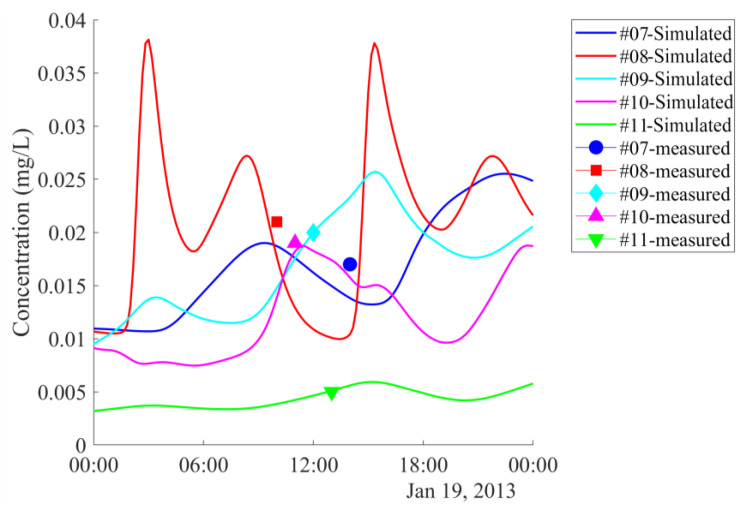
Simulated time course of concentration of chromium in water at stations 7–11 downstream of the Thi Vai river and measured data. Locations of these stations are shown in Figure 2.

By the procedure explained in Section 4.1, a parameter set was obtained which resulted in a good agreement between the simulation results and observations. Temporal variations of simulated Cr concentrations at the 11 stations (which were validated by measured data) along the Thi Vai River are shown in Figure 8 and Figure 9. As can be seen in Figure 8, high concentrations of Cr occurred at the stations close to the industrial zones (station one with a Cr concentration of 0.082 mg/L is near Go Dau and My Xuan industrial zones, station six with concentration 0.028 mg/L is near Phu My and Cai Mep industrial zones). The lowest Cr concentrations were found at station 11, located downstream near Ganh Rai Bay, as shown in Figure 9. The simulated concentrations were slightly lower than the measured data, but the trends were similar to those measured along the river.

Both measured and simulated concentrations increased from the onset of flood tide to high tide and decreased from ebb tide to low tide. The model reproduced this trend with NSE > 0.8, and d value > 0.9.

### 5.2. Scenarios Simulation Results

The model, after being calibrated and validated, was used to simulate three scenarios (described in Section 4.2 above) to examine the phytoremediation capacity of mangrove plants. The boundary conditions and initial conditions of the model were set based on the measured data of Cr concentrations in water, soil and plant. The running time was set from 1 January 2013 to 30 January 2013 so that it could cover the time of taking water and plant field samples.

#### 5.2.1. Chromium Concentration in Water

**Figure 10:**
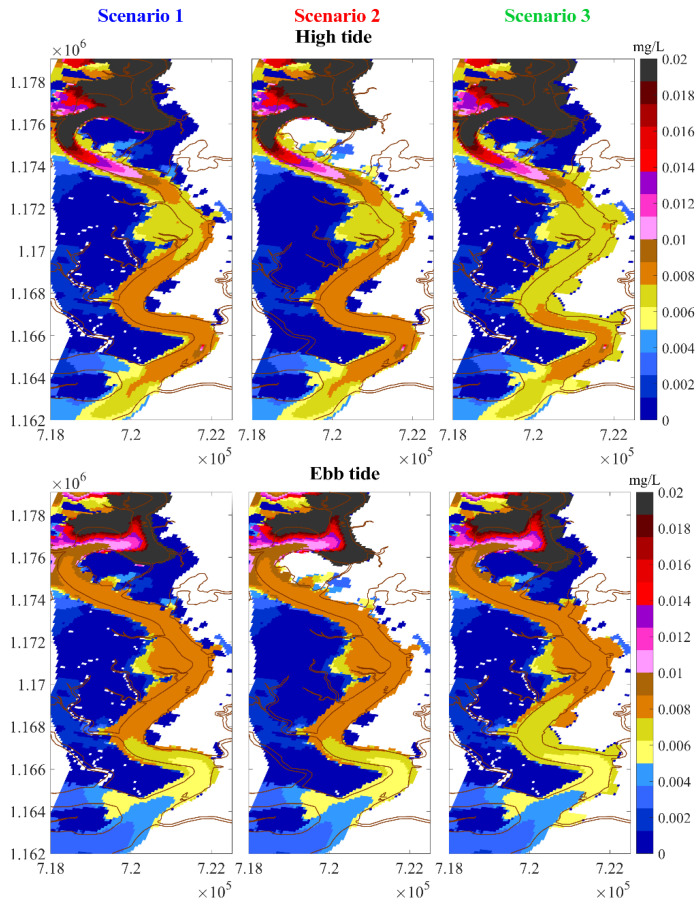
Concentration of chromium in water after one-month simulation.

**Figure 11:**
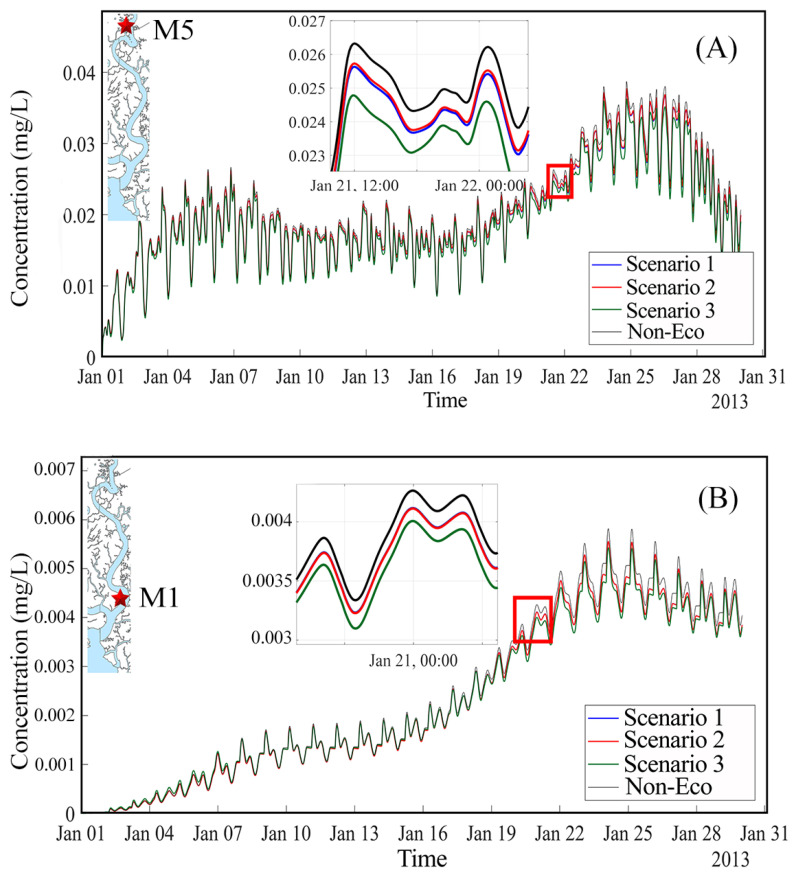
Cr concentration in water at the two stations at upstream (M5 as shown in (**A**)) and at downstream (M1 as shown in (**B**)).

The simulations shown in Figure 10 and Figure 11 clearly demonstrate the role of mangrove forests for the retention of heavy metals. In scenario two, Cr concentrations in water were just slightly higher than those in the baseline scenario. This explains that not all mangrove trees can take part in treating pollutants. The mangroves just take up pollutants when being exposed to polluted water and the trees growing on higher ground elevation do not uptake those substances in the water. Another reason is that the wastewater is directly discharged into the stream on the east bank without passing mangrove areas which are located on the west bank. If the wastewater is passed through artificial wetlands prior to discharge into the river, the role of mangroves for the retention of metals becomes quite clear, as shown in scenario three, where Cr concentration is much lower than those in the other two scenarios, as shown in Figure 10 and Figure 11.

An interesting effect is the increment trend of concentration of Cr in the upstream sections (Cr concentration at site upstream M5 got up to 0.04 mg/L and this value at site downstream M1 reached highest value at 0.006 mg/L), as shown in Figure 11. This is due to the characteristics of the tidal force in the area. The length of the river is too long, thus, wastewater is discharged to the river at neap tide and when it is on the way moving seaward, the tide comes from the ocean and pushes them back upstream. The time courses of Cr levels between the two measured stations at upstream (M5) and downstream (M1) are also different. Whereas concentrations are increasing at the downstream station M1, as shown in Figure 11B, they are fluctuating at the upstream station M5, as shown in Figure 11A. This is because the downstream area receives pollutants both from upstream sites and from adjacent sewage disposals. Furthermore, the time for pollutants transport to the sea is not long enough to release all pollutant concentration to the ocean. Subsequently, this area gets tidal water coming again from the sea. At upstream region, Cr concentration fluctuates by high water springs and low water neaps of tidal regime during the month, first increasing in approximately ten days (high water spring period) then decreasing in the next ten days (low water neap period) and after that it increases again, as shown in Figure 11A. However, both stations showed the increment trend of Cr concentration in the Thi Vai water. This also explains why the Thi Vai river was extremely highly polluted in the past and was marked as a “dead River” in 2004.

To analyze the effect of the soil–plant media on Cr concentration in water, we simulated one more scenario omitting the absorption processes of soil and plants. This scenario is called Non-Eco. Figure 11 shows Cr concentrations in water obtained from the four scenarios at stations M5 (upstream) and M1 (downstream), respectively. Cr concentrations increase at both stations. One can clearly distinguish between the four scenarios: Cr concentration levels are highest in the Non-eco scenario and lowest in scenario three, whereas the concentrations obtained in scenarios two and three lie in between.

#### 5.2.2. Chromium Concentration in Soil

**Figure 12:**
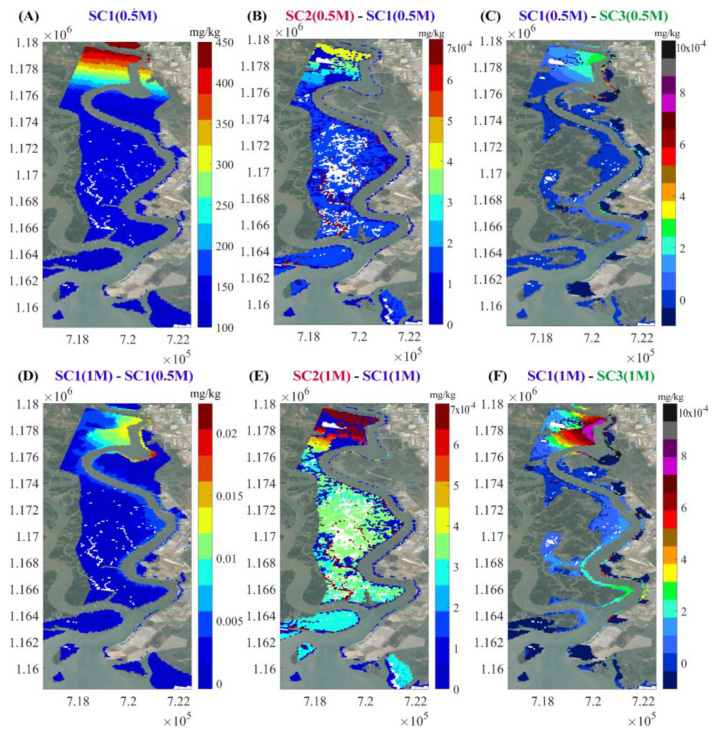
Distribution of chromium in soil after one month of simulation in three scenarios. Notations: (**A**) denotes the simulation result from SC1 in half a month (1M denotes simulation time in month (30 days) and 0.5M denotes simulation time in half a month (15 days)). (**B**) denotes the effect of the differences between SC2 and SC1 in half a month. (**C**) denotes the effect of the differences between SC1 and SC3 in half a month. (**D**) denotes the differences between SC1 in one month and SC1 in half a month. (**E**) denotes the effect of the differences between SC2 and SC1 in one month. (**F**) denotes the effect of the differences between SC1 and SC3 in one month.

The three scenarios are compared with respect to soil concentrations, as shown in Figure 12. Soil concentrations increased with time, as shown in Figure 12A,D with respect to SC1. Cr contents are mostly concentrated in the upstream regions, as shown in Figure 12D. Concentration in flooded areas is higher than in high elevation areas. Chromium concentration in the soil covered by mangroves is much lower than those without mangroves (comparison between scenarios one and two), as shown in Figure 12B,E. In scenario two, Cr is highly concentrated in soil because all mangroves were cut down. After 15 days, the difference in Cr concentration between SC2 and SC1 is up to 7 × 10^−4^ mg/kg/cell at the flooded areas and up to 4 × 10^−4^ mg/kg/cell in the upstream area. This difference increased after 30 days of simulation (up to 7 × 10^−4^ mg/kg/cell). In scenario three, the concentrations in the soil of artificial wetlands are higher than in the other two scenarios with bare wetlands and industrial infrastructures along the East bank, as shown in Figure 12C,F.

In all three scenarios, chromium concentration in the soil gradually increases upstream. The soil on the East bank of the Thi Vai River around the discharge sources accumulated the highest Cr concentration compared to other areas. Elevated soil concentrations are correlated with elevated water concentrations, as shown in Figure 10. Chromium concentration is high in the soil at flooded areas. The most important feature is that afforestation, as with setting up artificial wetlands with mangroves at the discharge locations, reduces the soil pollution in natural forest on the opposite area, as shown in Figure 12C,F.

**Figure 13:**
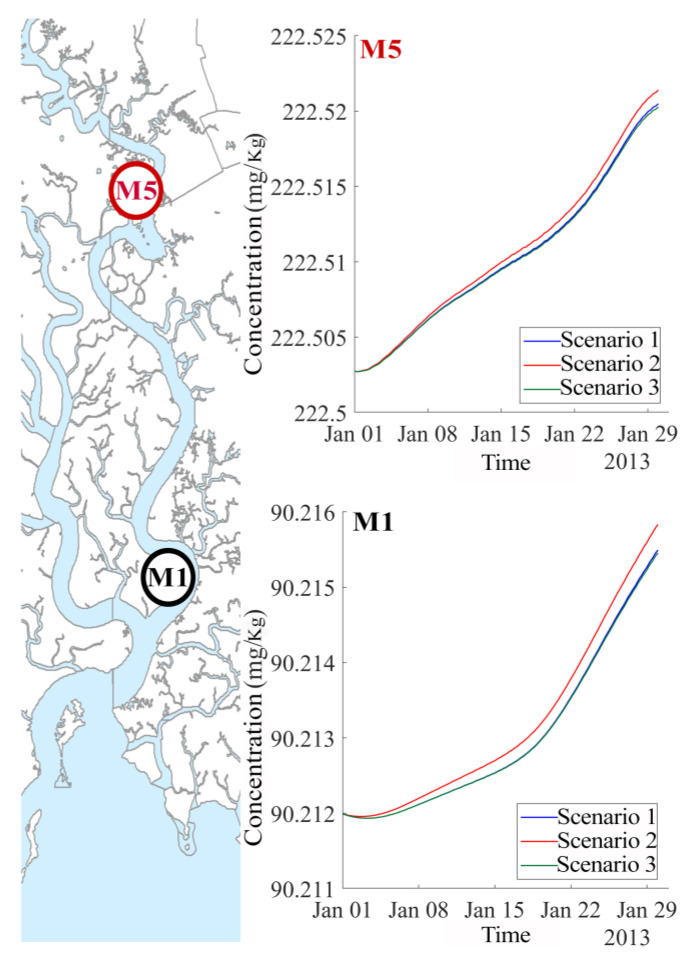
Chromium concentration at representative stations at upstream (M5) and downstream (M1).

Chromium concentrations (both in solid phase and in liquid phase) in the soils at the two stations M5 (upstream) and M1 (downstream) increase slightly after one month of simulation. The upstream area has higher concentration than that in the downstream area, as shown in Figure 13 (the concentration values are approximately 223 mg/Kg/cell at M5 and 90 mg/Kg/cell at M1). It is interesting to note that concentrations are rising after a lag phase, as can be seen at station M1 due to the tide forces.

#### 5.2.3. Chromium Concentration in Mangrove Trees

**Figure 14:**
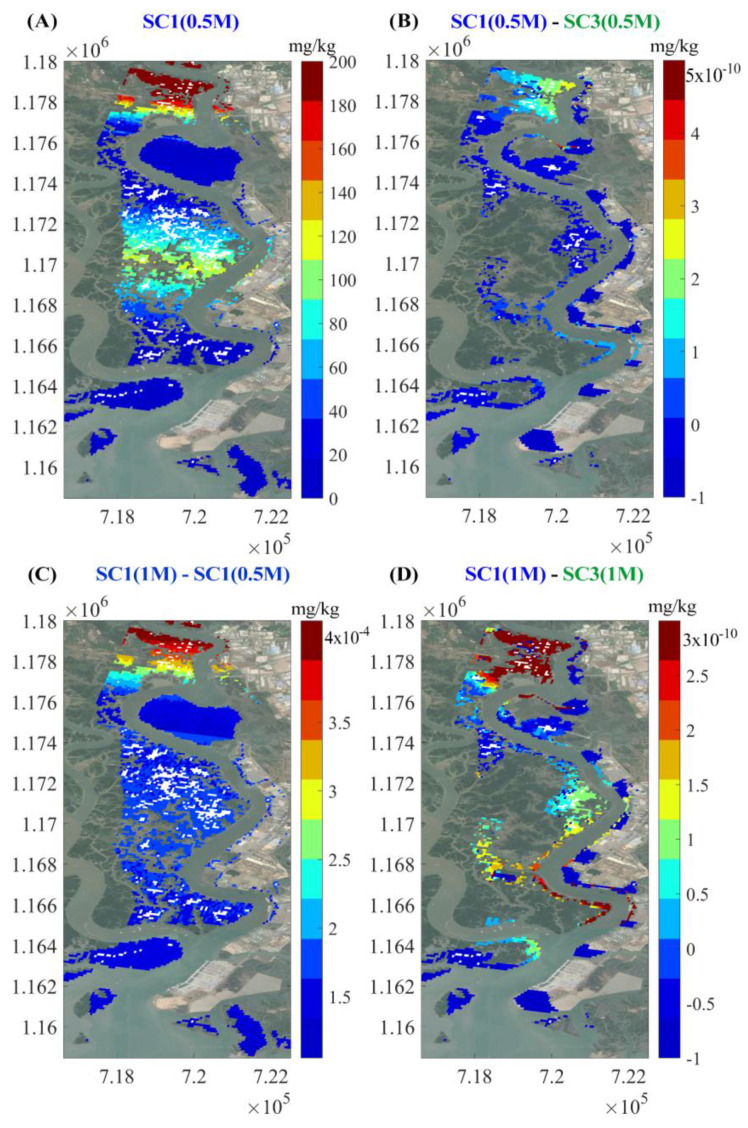
Distribution patterns of chromium in mangroves in different scenarios (**A**) shows the concentration patterns in trees under scenario one after 0.5 months. (**B**) shows the pattern of the concentration differences between scenario one and scenario three after a simulation time of 0.5 months. (**C**) shows a map of the differences between scenario one at 1 month and scenario one at 0.5 months. (**D**) shows a map of the differences between scenario one at 1 month and scenario three at 1 months.

**Figure 15:**
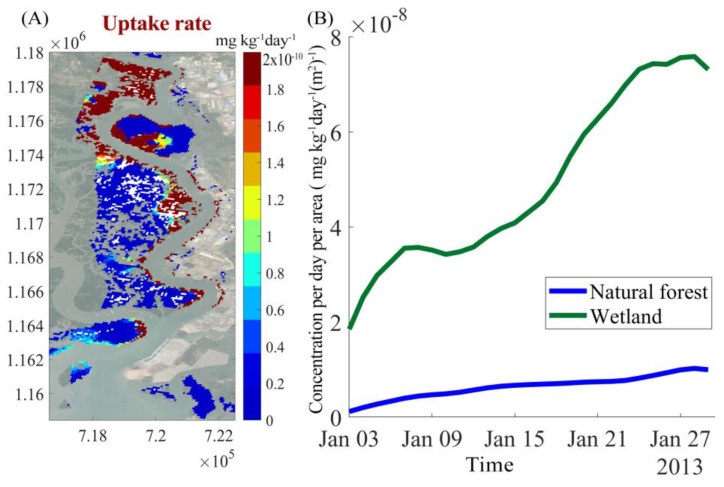
(**A**) The spatial distribution of tree uptake rates (mg/kg/day). (**B**) Uptake rates of Cr by mangroves in artificial wetlands on the east bank and by natural forests on the west bank (mg/(kg·day·m^2^)).

The simulations show that the accumulation of Cr in mangrove trees is highest in scenario three since, in this scenario, more mangrove trees were planted along the industrial locations on the East bank of the river. The upstream forest accumulates more Cr than the forests in the middle and downstream sections. Chromium concentration in trees in SC1 after 15 days gets up to 200 mg/kg/cell at upstream areas, 120 mg/kg/cell at middle stream areas and 20 mg/kg/cell at downstream areas, as shown in Figure 14A. The increment of Cr from day 15 to day 30 is highest at upstream region (increasing up to 4 × 10^−4^ mg/kg/cell), as shown in Figure 14C. The artificial wetlands in SC3 demonstrated a high capacity in absorbing Cr. This effect can be observed by differences in Cr concentrations between SC1 and SC3, as shown in Figure 14D. This is also reflected by the uptake rates, which reached about 2 mg/kg/day in the upstream section, as shown in Figure 15A.

Figure 15 compares the Cr uptake rates of mangroves in the artificial wetlands with the Cr uptake rates of mangroves at the opposite riverbank. The differences are large—uptake rates within the constructed wetlands are much higher than those of natural forest, as shown in Figure 15B. The reason is that mangroves in the artificial wetlands are at an optimal age (young mangrove plants) for substance uptake. We applied the parameter k_13_ (which expresses the rate of taking up water and matter from soil to tree, as shown in Eq. 3), estimated from the experimental data of Richter et al. [20] for young trees (k_13_ = 7 × 10^−8^). However, this value had to be decreased in the tuning for the actual field situation, which is characterized by a mature even aged mangrove forest (as described in Section 4.1). The lower value of k_13_ of mature plant leading to less matter (including Cr) and water transport to the plant can be explained by their root systems. A root system consists of a complex network of individual roots that vary in age along their length [53]. Fine roots are the most permeable portion of a root system and are thought to have the greatest ability to absorb water. Roots of woody plants form bark as they age, much like the trunks of large trees. The bark formation decreases the permeability of older roots [53,54,55]. In addition, the wetlands are located directly at the discharge sources, whereas Cr concentrations are lower at the west bank opposite the disposal sites. So, the large differences are explained both by a lower value of k_13_ and a higher chromium load in the artificial wetlands.

### 5.3. Model Applicability and Further Development

Results from running three scenarios have proven the important role of mangrove trees in reducing pollution. The total constructed wetlands area that was built in SC3 was about 3 km^2^ and the total area of the study region was about 172 km^2^. The addition of about 1.7 % area of mangrove trees into the study region at appropriate locations can help to decrease the total amount of pollutant—up to 33 kg chromium every month for the whole study area (this is calculated from the summation of the contents accumulated in artificial wetlands on the East bank, as shown in Figure 10). These results reveal a promising potential of mangrove forests in treating industrial wastewater and the role of constructed mangrove wetlands in reducing the amount of heavy metal pollutants. Those plants growing in constructed wetlands can be easily collected and treated later.

To our knowledge, there is no similar study conducting a simulation of the phytoremediation process on a landscape scale except the previous publication of Nguyen and Richter [27]. However, this model was at the theoretical stage and was not calibrated for a real situation. Although the model presented here is capable of simulating realistic scenarios, its application is limited to planted even age forests and to the metal chromium. In order to broaden the scope of model applications, reliable estimates of the parameters governing the uptake of metals (the parameter k_13_ in Eq. 3) at different tree ages and in different soil types are necessary. This requires an adequate experimental design with factors plant age, soil conditions and heavy metal contents.

Concerning the hydrodynamic part of the model, there is still much uncertainty on Manning’s coefficients for mangroves of different age classes and densities. However, we have succeeded in coupling the hydrodynamic model Delft3D, which gave excellent results for the time courses of water levels and discharges in the Thi Vai river, with an aggregated environmental fate model for Cr. From a technical point of view, this framework can be easily extended to integrate environmental fate models of higher resolution concerning the incorporation of plant organs, such as roots, stems and leaves.

## 6. Conclusions

Simulation results for the case of the Thi Vai catchment showed the applicability of our integrated model to the analysis, calculation and allocation of mangrove afforestation areas in appropriate locations to promote the role of their phytoremediation potential. The simulation tool presented in this paper is capable of supporting environmental managers in their difficult task to protect the environment in balance with economic development. The problem is, on the one hand, mangroves have an important role and function as a natural protection system (natural phytoremediation system); however, on the other hand mangrove ecosystems themselves are highly sensitive to all kinds of disturbances. Therefore, the next step should be to identify the intrinsic thresholds and impact factors in which the role of mangrove forests can be promoted under the influences of extreme conditions. In the case the impact exceeds the bearing capacity of the mangroves, degradation trends of mangrove ecosystems have to be predicted. This will help the managers to devise protection plans and to regulate the reforestation of mangrove forests in the future. Thus, further model developments should imply forest dynamics to predict long term developments of mangrove forests under changing environmental conditions.

## Figures and Tables

**Table 1:** Wastewater discharge from industrial zones at the Thi Vai River (data based on the investigation of the Institute for Environment and Resource in 2008).

Discharge Source	Cr Discharge (mg/s)	Discharge Period
A2	0.5	Wastewater is being discharged at neap tide (at about 12:00–14:00 every day)
A1	1	
Cai Mep	1	
Phu My	0.2	
Go Dau	2	

Note: the positions of discharge sources are shown in Figure 7.

**Table 2:** Model performance of the calibration and validation processes.

Water Level		Dry Season			Rainy Season		
		NSE	d	RMSE (m)	NSE	d	RMSE (m)
Calibration	Station 1	0.816	0.953	0.345	0.937	0.984	0.259
	Station 4	0.86	0.964	0.303	0.867	0.965	0.402
Validation	Station 2	0.695	0.897	0.998	0.962	0.99	0.208
	Station 3	0.865	0.965	0.309	0.946	0.986	0.252
